# Mental health and quality of life in children and adolescents during the COVID-19 pandemic: a systematic review of longitudinal studies

**DOI:** 10.3389/fpubh.2023.1275917

**Published:** 2024-01-08

**Authors:** Ester Orban, Lydia Yao Li, Martha Gilbert, Ann-Kathrin Napp, Anne Kaman, Sabine Topf, Maren Boecker, Janine Devine, Franziska Reiß, Flora Wendel, Caroline Jung-Sievers, Vanessa Sophie Ernst, Marco Franze, Eva Möhler, Eva Breitinger, Stephan Bender, Ulrike Ravens-Sieberer

**Affiliations:** ^1^Department of Child and Adolescent Psychiatry, Psychotherapy and Psychosomatics, University Medical Center Hamburg-Eppendorf, Hamburg, Germany; ^2^Department of Child and Adolescent Psychiatry, Child Neuropsychology Section, University Hospital RWTH Aachen, Aachen, Germany; ^3^Faculty of Medicine, Institute of Medical Information Processing, Biometry and Epidemiology (IBE), Ludwig-Maximilians-University Munich, Munich, Germany; ^4^Pettenkofer School of Public Health, Munich, Germany; ^5^Institute for Community Medicine, Epidemiology of Health Care and Community Health, University Medicine Greifswald, Greifswald, Germany; ^6^Child and Adolescent Psychiatry, Psychosomatics and Psychotherapy, Saarland University Medical Center, Homburg, Germany; ^7^Department of Child and Adolescent Psychiatry, Psychosomatics, and Psychotherapy, Faculty of Medicine and University Hospital Cologne, University of Cologne, Cologne, Germany

**Keywords:** systematic review, children, adolescents, mental health, quality of life, COVID-19, prospective studies

## Abstract

**Background:**

The COVID-19 pandemic has significantly impacted the mental health of children and families, i.e., due to measures like social distancing and remote schooling. While previous research has shown negative effects on mental health and health-related quality of life (HRQoL), most studies have focused on pre-post comparisons in the early pandemic stages. This systematic review aims to examine longitudinal studies to understand the long-term impacts of the pandemic on children and adolescents.

**Methods:**

This systematic review adhered to the PRISMA guidelines and was preregistered in the international prospective register of systematic reviews (Record ID: CRD42022336930). We systematically searched PubMed/MEDLINE, Web of Science, PsycINFO, PSYNDEX, and the WHO-COVID-19 database and included studies published up to August 30, 2022. Based on pre-defined eligibility criteria, longitudinal and prospective studies that assessed the mental health or quality of life of children or adolescents (0–19 years) in the general population over a longer time span (at two or more measurement points) during the COVID-19 pandemic were included in the review. The methodological quality of the included studies was assessed using an adapted version of the Effective Public Health Practice Project (EPHPP) checklist. Narrative data synthesis was used to summarize the findings.

**Results:**

A total of 5,099 results were obtained from literature searches, with 4,935 excluded during title/abstract screening. After reviewing 163 full-text articles, 24 publications were included in the review. Sample sizes ranged between *n* = 86 and *n* = 34,038. The length of the investigated time periods and the number of assessment points, as well as outcomes, varied. The majority of studies were of moderate methodological quality. Mental health outcomes were more frequently studied compared to measures of HRQoL. The findings from these studies mostly suggest that children and adolescents experienced heightened mental health problems, specifically internalizing symptoms like anxiety and depression. Further, there was a decline in their overall HRQoL over the course of the COVID-19 pandemic that did not necessarily subside when lockdowns ended.

**Conclusion:**

It is crucial to continue monitoring the mental health and well-being of children and adolescents following the pandemic to identify groups at risks and plan interventions. This should ideally be conducted by large systematic studies, using validated instruments, and encompassing representative samples to obtain reliable and comprehensive insights with the aim of improving youth mental health care.

## Introduction

The COVID-19 pandemic has significantly interfered with the daily lives of children and families. Although the direct physical health effects of the coronavirus infection appear to be minor in the young population (1, 2), children and adolescents may suffer severely from the indirect effects of the pandemic on mental health. Pandemic containment measures such as social distancing and restrictions on social gatherings, lockdowns, and phases of complete or partial home and online schooling have limited children’s and adolescents’ possibility of socializing and engaging in physical activity or play. Peer interaction, which is an important aspect of development, has been limited (3). Various studies have shown that the mental health and well-being of children and adolescents have been negatively affected during the pandemic. For instance, symptoms of depression and anxiety have increased compared to the pre-pandemic state (4, 5). This has been observed in adults as well, but the younger population appears to be particularly vulnerable (6). Studies have noted an initial reduction in provision and use of child and adolescent psychiatric services in the early phase of the pandemic (7, 8), while providers signaled a substantial increase in the number of referrals and requests for assessments 1 year after the start of the pandemic (9). The initial reductions in youth psychiatric service provision indicate delays or unmet needs early in the pandemic, and alarmingly some evidence points at increased suicide rates in the second wave of the pandemic (July to October 2020) (8). This is obviously of serious public health concern, also because mental health issues in childhood are associated with an elevated risk of adult mental disorders (10).

A vast amount of research on child mental health has been published since the beginning of the COVID-19 pandemic and the body of evidence is constantly evolving. Several reviews on mental health and quality of life in children and adolescents have predominantly identified evidence of a negative impact of the pandemic (4, 11–22).

The majority of reviewed original studies however relied on cross-sectional data. The need for longitudinal mental health research in the young population was identified early in the pandemic (23). Systematic reviews and meta-analyses of longitudinal studies on the psychological impact of the COVID-19 lockdown have been published, but predominantly included studies on adults (24, 25). Further, most longitudinal research has focused on comparing pre-pandemic outcomes with outcomes measured after the start of the pandemic (26), mostly at a single pandemic time point. In their recent systematic review on this type of pre-post COVID-19 studies, Kauhanen et al. (22) found a predominantly negative impact on mental health in adolescents and young people, particularly increased depression, anxiety and psychological distress. Studies with pre-pandemic and pandemic data were also analyzed in a meta-analysis by Ludwig-Balz et al. (27), focusing on depressive symptoms in young Europeans. The review reported an increase in depressive symptoms, while evidence for clinically relevant depression was of low certainty (27). The same authors found in another recent meta-analysis an increase of anxiety symptoms during school closures in Europe (28). Another systematic review and meta-analysis with a similar focus was published by Newlove-Delgado et al. (21). The studies included in these reviews mainly refer to the early phases of the pandemic in the first half of 2020.

Now, over 3 years since the pandemic began, we wonder how children have been faring throughout this period. In this context, longitudinal studies aiming to assess COVID-19-related mental health trajectories have started to emerge. However, to the best of our knowledge, a systematic synthesis of evidence from longitudinal studies, focusing on children’s long-term mental health or quality of life trajectories during the pandemic using at least two pandemic assessment points, has not been published to date.

Therefore, our objective is to focus beyond pre-post comparisons and conduct a systematic review of longitudinal studies on mental health and quality of life outcomes in children and adolescents during the COVID-19 pandemic, specifically focusing on general population studies with multiple data assessment points covering longer periods during the pandemic. The review aims to address the key question of how the mental health and quality of life of children and adolescents in the general population have developed over the course of the COVID-19-pandemic. It is important on a public health scale to assess whether long-term consequences for children’s mental health and well-being persist, also considering the management of future similar crises that might emerge.

## Methods

The procedure and reporting of this systematic review are in line with the recommendations of the Preferred Reporting Items for Systematic Review and Meta-Analysis (PRISMA) guidelines (29). The review protocol was published *a priori* in the international prospective register of systematic reviews (PROSPERO) on June 6, 2022 (Record ID: CRD42022336930, https://www.crd.york.ac.uk/PROSPERO/display_record.php?RecordID=336930).

### Eligibility criteria

Inclusion and exclusion criteria are based on the PECO scheme (population–exposure–comparison–outcome) (30) and were determined *a priori.*

#### Population

Original studies on children and adolescents aged 0–19 years were included in this review. We refer to the definition of adolescents (10–19 years) by the World Health Organization (WHO) for the upper age limit (31). We excluded studies that focused on older individuals, studies that did not report the age of the included subjects, or studies on a broader age group including ages 0–19, but not reporting subgroup results for ages 0–19.

Population, community or school-based studies were included. We excluded studies with a focus on clinical populations or participants that were sampled or studied for specific health conditions, as our aim was to study the general population.

#### Exposure

To be included, studies must have measured a relevant outcome on at least two occasions *during the COVID-19 pandemic.* We defined the start of the pandemic as after March 11, 2020 (i.e., the date the WHO declared the pandemic), and for Chinese studies after January 23, 2020, when substantial contact restrictions were put in place by the Chinese government.

#### Comparison

Included studies needed to report a comparison of at least two outcome assessments during the COVID-19 pandemic (i.e., after the start of the pandemic). “Comparison” refers to a statistical analysis difference/change in outcome between assessment points, including a reported estimate and/or value of *p*. We included any kind of effect measure reported for these comparisons. Reports of descriptive data without statistical testing were excluded.

#### Outcomes

The outcomes of interest are self- or proxy-reported measures of mental health or (health-related) quality of life in children/adolescents. These primarily comprise results from screening tools and rating scales like the Strengths and Difficulties Questionnaire (SDQ), Center for Epidemiologic Studies Depression Scale (CES-D), the KIDSCREEN, etc. The same instrument had to be applied at all compared assessment points. Examples of mental health outcomes include depressive symptoms, symptoms of anxiety, internalizing/externalizing symptoms, behavioral problems, and stress.

#### Study design

This systematic review included any type of longitudinal/prospective studies that used surveys or interviews to determine the mental health or quality of life of children or adolescents at multiple assessment points. These included cohort, repeated cross-sectional, panel, time series, and time trend studies. We also included studies that compared samples from different surveys if they demonstrated that the populations were comparable. We excluded cross-sectional studies without follow-up, experimental studies, and intervention studies which mainly focused on intervention effects.

As this review focuses on long-term trajectories of mental health beyond the initial phase of the pandemic, we only included studies that cover a period of at least 6 months during the pandemic, meaning the time between the first and last outcome assessment after the above-mentioned start dates. Studies covering a pandemic time period of <6 months and studies that only compare outcomes before and after the beginning of the pandemic (with only one time point after) were excluded.

We further excluded duplicate publications of results from the same study/population. In this case, we included the study that provided the most information regarding our research questions.

#### Publication type

Studies were eligible for inclusion if they were peer-reviewed publications reporting original study results; other publication types, such as reviews, letters to the editor, opinion papers, conference abstracts and preprints, were excluded. We only included studies published after 03/2020. We did not limit the publication language; however, our search terms were in English.

### Data sources and search strategy

The first two authors (EO, LL) searched PubMed/MEDLINE, Web of Science, PsycINFO, PSYNDEX, and the WHO-COVID-19 database on August 30, 2022. Based on the pre-defined eligibility criteria and the PICO framework, we used a combination of search terms referring to the population (“child* OR adolescent* OR youth OR pediatric* OR infant*”), COVID-19 pandemic (“COVID-19 OR coronavirus OR sars-cov-2 OR pandemic OR lockdown OR school closure”), outcomes (“mental health OR well-being OR depressi* OR anxi* OR psycholog* OR stress OR mental distress OR PTSD OR loneliness OR internalizing OR quality of life OR QoL OR HRQoL”), and study type (“longitudinal or prospective or cohort or trajector*”), which were then adapted to the respective database. The full search strategy can be found in the Appendix 1. We also searched Google Scholar, checking the first 200 results. As we aimed to identify studies conducted in the context of the COVID-19 pandemic, searches were limited to studies published after the beginning of 2020.

### Study selection

After deduplication, titles and available abstracts of the retrieved records were screened for eligibility by the reviewers (EO, LL, MG, A-KN, VE, MF, and EB). We piloted the title/abstract screening process on 50 records that were each screened by two reviewers independently. Given the very high degree of agreement between the two reviewers, it was decided that double screening of all the records was not necessary at this stage.

In the second step, two reviewers each (EO, LL, MG, A-KN, FW, VE, MF, and EB) independently screened the full texts of the included records. Disagreements between reviewers were discussed until consensus was reached, involving a third party if necessary. Reference lists of included studies and identified relevant reviews were screened for further potentially eligible publications (MG, A-KN).

EndNote was used to collect and de-duplicate the records. For the screening of titles and abstracts, we used the web-based application Rayyan.^1^ At the full text screening stage of the screening process, we documented the reasons for exclusion using a Microsoft Excel spreadsheet. Reasons of exclusion were documented in hierarchical order, meaning that in case of multiple reasons for exclusion the first reason (publication type > population > outcome > study design > comparison/pandemic time points) was documented.

### Data extraction process and synthesis method

Study characteristics and study data were extracted independently by teams of two reviewers (EO, LL, FW, MF, VE, and EB) using a standardized spreadsheet. The following information was extracted: First author and year, country, research question, study design, times of data collection, sample size, age of participants, information on sample and setting (e.g., from which study, general or other population, gender and distribution), caregiver age and gender (if applicable), outcomes, instruments used to measure outcomes, statistical methods, and results (see Table 1).

**Table 1:** Rating of methodological quality (risk of bias) of the included studies, in alphabetical order.

First author, year	Selection bias	Study design	Detection bias	Attrition bias	Statistical methods	Overall quality
Adachi et al. (45)	M	H	H	H	H	H
Albrecht et al. (37)	L	M	H	L	H	L
Cimino et al. (38)	L	H	H	L	H	L
Fischer et al. (36) (KLIK)	M	M	H	L	M	M
Fischer et al. (36) (NTR)	L	M	H	L	M	L
Gordon-Hacker et al. (54)	L	H	H	M	H	M
Hafstad et al. (39)	H	M	H	L	H	M
Hagihara et al. (46)	M	H	H	L	H	M
Lehmann et al. (34)	L	H	H	L	H	L
Lehmann et al. (35)	L	H	H	L	H	L
Lengua et al. (50)	L	H	H	M	H	M
Martinsone et al. (40)	L	H	H	M	H	M
Nikolaidis et al. (53)	M	H	L	L	H	L
Poulain et al. (41)	L	H	H	M	H	M
Ravens-Sieberer et al. (5)	M	H	H	M	H	H
Raymond et al. (51)	L	H	H	M	H	M
Theuring et al. (42)	M	H	H	H	H	H
van der Laan et al. (43)	M	H	H	L	H	M
Weissman et al. (52)	L	H	H	H	H	M
Wenter et al. (44)	L	M	H	L	H	L
Westrupp et al. (56)	H	H	H	M	M	M
Xie et al. (47)	M	H	H	H	M	H
Zhang et al. (48)	L	H	H	H	M	M
Zhou et al. (49)	M	M	H	L	M	M
Zuccolo et al. (55)	M	H	H	L	M	M

L, Low; M, Moderate; and H, High quality rating.

A meta-analysis was not conducted since the included longitudinal studies applied especially heterogeneous outcome assessments and statistical methods. Therefore, the reported effect measures varied highly. Furthermore, the assessed time periods and variety in pandemic protection measures imply different circumstances during the pandemic. The study results were thus narratively synthesized. The following main aspects were considered to organize and synthesize the study results systematically: type of outcome (i.e., internalizing symptoms, externalizing symptoms, and quality of life), gender differences, study size, covered time periods, and whether there was a pre-pandemic outcome measurement. The study periods and number of time points were summarized visually in the results section.

### Risk of bias assessment

The methodological quality (risk of bias) was assessed in all included studies using an adapted version of the Effective Public Health Practice Project (EPHPP) checklist (32, 33) (see Appendix 2). Summarizing five subdomains (selection bias, study design, detection bias, attrition bias, and statistical methods), studies received an overall rating of low, moderate or high quality. Risk of bias in studies was assessed independently by teams of two reviewers (rating reviewers: EO, MG, and A-KN). Reporting bias was assessed indirectly through the EPHPP checklist, which considered whether relevant information was reported.

## Results

**Figure 1:**
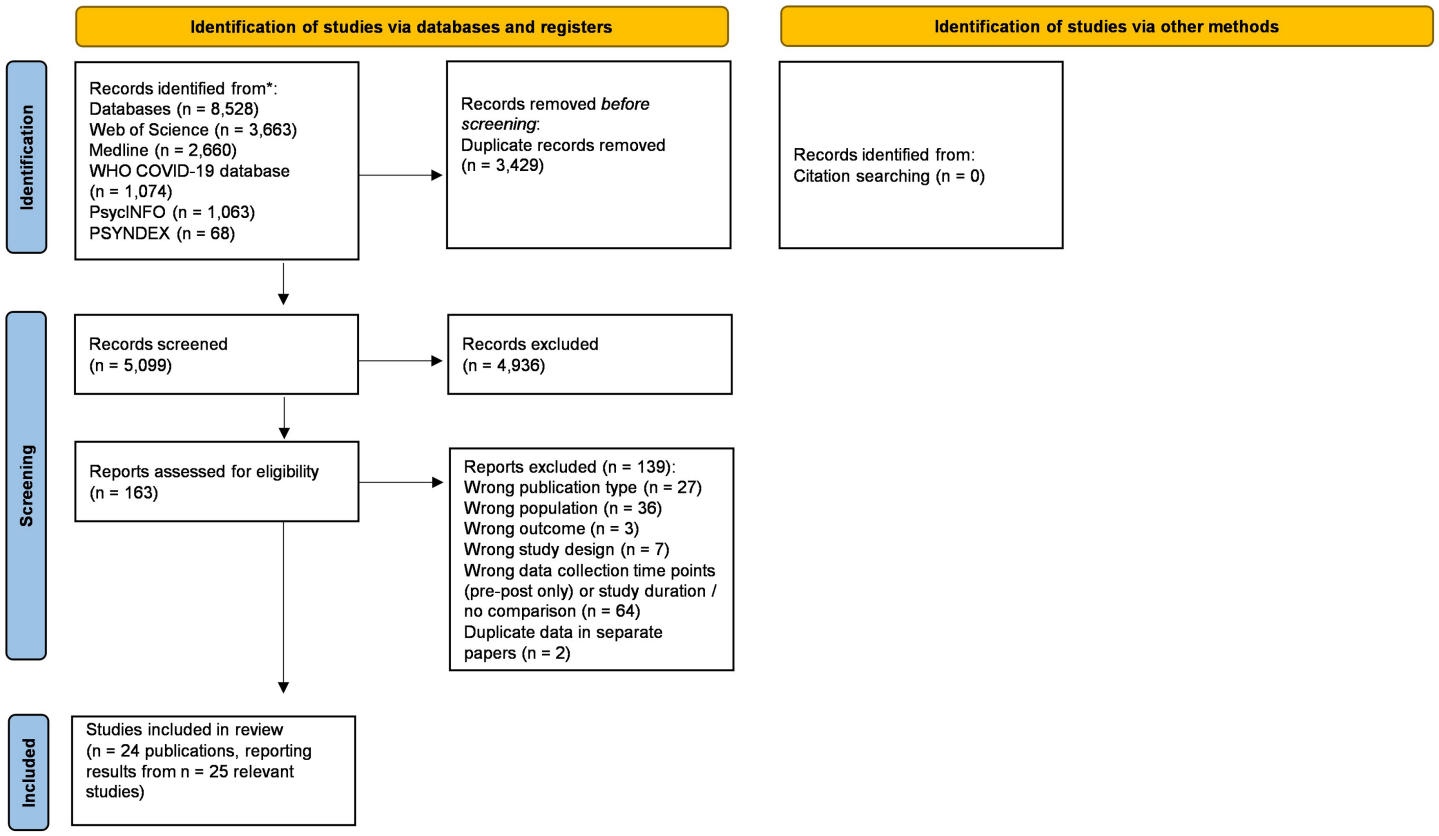
PRISMA flow chart of the systematic review process (57).

Figure 1 shows the PRISMA flow chart of study identification and selection. The literature searches generated a total of 5,099 results after removal of duplicates, of which 4,936 were excluded at the title/abstract screening stage. After screening the remaining 163 full-text articles, 24 articles were included in the review, covering a total of *n* = 24 studies. Notably, two of the included articles reported results from the same study population (but different outcomes) (34, 35), and one article reported relevant results from two different study populations separately (36).

Important study characteristics are summarized in Table 1. The 24 included studies comprised populations from 16 different countries, with sample sizes ranging from *n* = 84 to *n* = 34,038. Thirteen (54%) of the studies included >1,000 participants. The majority of the studies were conducted in Europe (*n* = 12) (5, 34–44). Some studies were from East Asia (*n* = 5) (45–49) and very few from the United States/Canada (*n* = 3) (50–52). One study included both United States and United Kingdom populations (53), and there were single studies conducted in Israel (54), Brazil (55), and Australia (56).

Most studies focused on school-aged children or adolescents (~7–19 years) and relied on self-reported outcomes, only four studies focused on children younger than 7 years and all of them used caregiver-reported outcomes (38, 44, 46, 54). All studies included both male and female participants, with the proportion of girls ranging from 43.7 to 67.5%.

**Figure 2:**
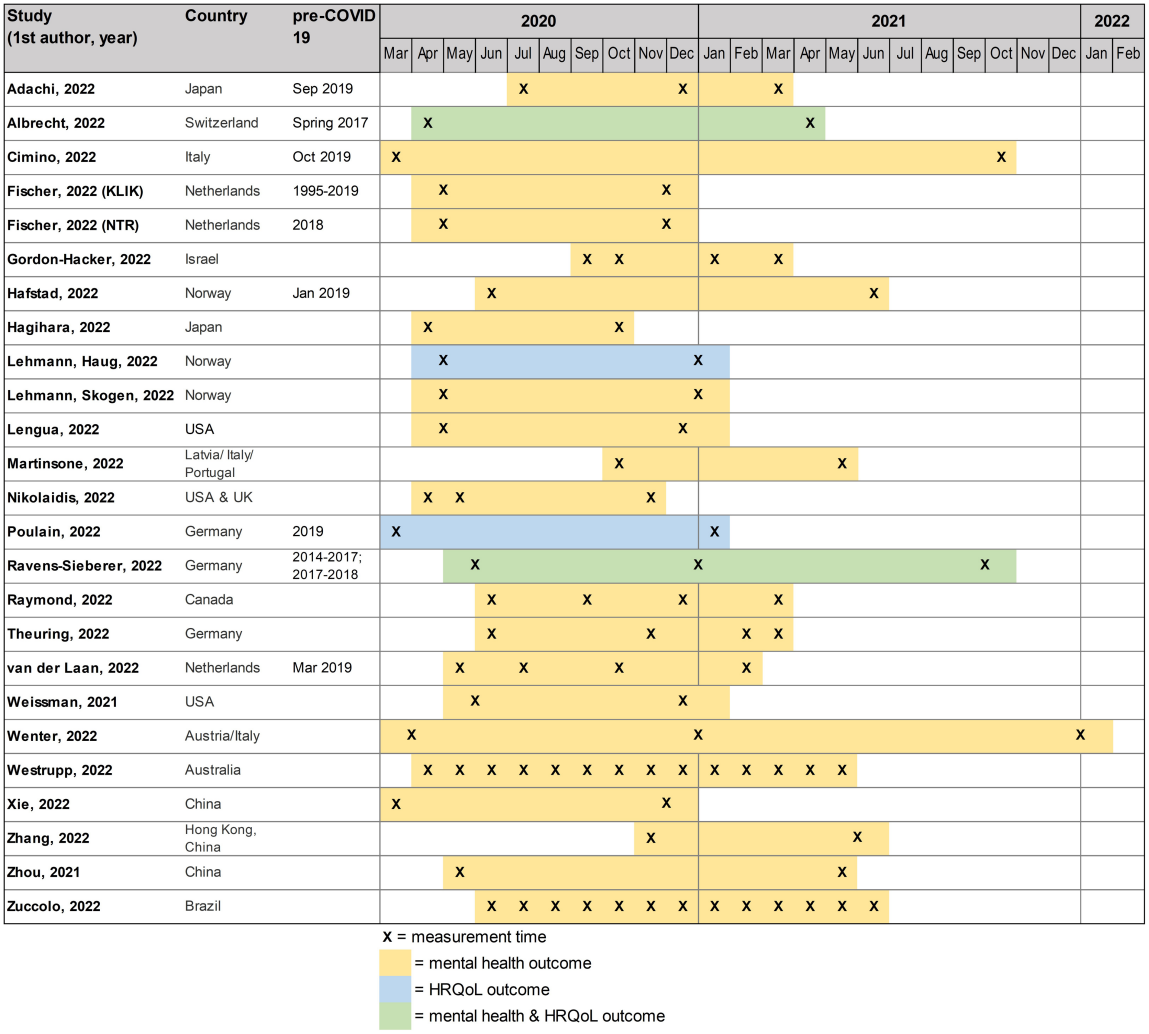
Overview of the timelines of the included studies, in alphabetical order.

The majority of studies were of longitudinal design (*n* = 18) (5, 34, 35, 37, 40–43, 45–48, 50–56); six were repeated cross-sectional studies with completely or largely different study subjects (36, 38, 39, 44, 49). Length of investigated time periods and number of assessments varied (see Figure 2). Most studies had two assessment points during the pandemic (range 2–14); one study reported measuring every 2 weeks for about 12 months (55). Eight studies additionally included a comparable pre-pandemic outcome measure (5, 36–39, 43, 45). The pandemic time periods covered by the studies ranged from 6 to 21 months (mean = 10.2 months) and the data was from 2020 and 2021 (Figure 2).

Considering the outcome domains of interest, mental health outcomes (*n* = 23 studies) were more frequently studied (5, 35–40, 42–56) than HRQoL (*n* = 4), which was measured using the KIDSCREEN in all identified studies (5, 34, 37, 41). The most frequently investigated mental health outcomes were depressive symptoms (*n* = 12) and anxiety symptoms (*n* = 10). The assessment instruments varied between studies, depressive symptoms were measured using the Patient Health Questionnaire (PHQ), Revised Child Anxiety and Depression Scale (RCADS), or the Child Depression Inventory (CDI); anxiety symptoms were, e.g., measured by the Generalized Anxiety Disorder Scale (GAD-7) or by the Screen for Child Anxiety Related Disorders (SCARED), to give a few examples. Further investigated mental health outcomes mainly comprised broader constructs of internalizing and/or externalizing symptoms, mostly measured through the SDQ (*n* = 7). Many of the studies investigated multiple outcomes (see Table 1).

Based on the criteria of our risk of bias assessment tool (Appendix 2), we identified studies of high (*n* = 4), moderate (*n* = 14), and low (*n* = 7) quality (see Table 1).

### Mental health—internalizing symptoms

#### Depression

The studies investigating measures of depressive symptoms (*n* = 12) covered a median of 9 months (range: 7–19 months) pandemic study time (see Figure 2). Five of the studies also included a pre-pandemic measure of depressive symptoms (5, 36, 38, 39, 45). Of these studies, four showed increased depressive symptoms during the pandemic compared to before (5, 36, 38, 39). In two of the studies, the pre-post increase in depressive symptoms was observed only later during the pandemic, namely in July 2021 in Norway (UEVO study) with no increase observed initially in June 2020 (39), and during the second lockdown in Germany (December 2020, COPSY study) (5). Only one study found decreased depressive symptoms during compared to before the pandemic (between Sept 2019 and Jul 2020). However, this was only observed in a group of children without smartphones; there was no change in the other group (45).

Considering the trajectories of depressive symptoms during the pandemic, the study results are mixed. Most studies (*n* = 7) found that depressive symptoms increased (or remained elevated compared to pre-pandemic levels) during the pandemic in the studied populations (36–39, 47, 51, 55). In a smaller study in Italy on children aged 5–6 years, Cimino et al. (38) surprisingly only found an increase in the group of children with mothers not at risk of psychological problems, while there was no change in depressive symptoms in the at-risk group.

Other studies observed fluctuating trajectories, with peaks related to periods of high infections and lockdowns (5, 56). A large Australian study included mothers of children aged 0–18 and applied a unique design with 14 assessment points, comparing a state with strict second wave lockdown in Victoria to states with no lockdown (56). They found no state differences during the first national lockdown (Apr/May 2020), but another peak in Victoria during their second lockdown (Jul–Oct 2020), that later subsided and was not observed in the states with looser restrictions. The German COPSY study (5) examined a large representative community sample of children aged 7–17 years in a longitudinal design, including pre-pandemic data and comparing this with three pandemic assessment points. They similarly observed an increase in depressive symptoms during the second infection wave/lockdown (Dec 2020/Jan 2021), which returned to pre-pandemic levels after restrictions were loosened (5).

Four of the studies noted a decline of depression symptoms throughout the pandemic (36, 45, 48, 49). A big study of Japanese children 9–12 years of age noted a decrease over three pandemic assessment points between July 2020 and March 2021, with higher symptoms before the pandemic, however only in the group of children without smartphones. They also found that depressive symptoms were higher in smartphone-owning children compared to non-owners (45). The Dutch KLIK study including 8–18 year-old also found that depressive symptoms decreased after lockdown (Nov/Dec 2020) compared to during lockdown (Apr 2020), but it is important to note that symptoms were higher than pre-COVID at both assessment points during the pandemic (36). Two Chinese studies noted a decrease in depression symptoms by May/June 2021 compared to earlier phases of the pandemic (48, 49). One study was conducted on a big sample with a mean age of 16.4 years (49), the other was a smaller study with children aged 9–11 years (48); neither of them included a pre-pandemic comparison. The study by Zhang et al. (48) did not cover the early pandemic period, as the first assessment was in November 2020.

In summary, study results were mixed, but a strong majority of the evidence points toward a continued increase in depressive symptoms after the beginning of the pandemic and a correlation of higher symptoms during times of higher infections rates and/or pandemic restrictions such as lockdowns. Further, where pre-pandemic data were available, all studies except one noted an increase in depressive symptoms after the beginning of the pandemic.

#### Anxiety

Nine of the 12 studies that investigated depressive symptoms also measured anxiety symptoms (5, 36, 39, 47–49, 51, 55, 56). One additional longitudinal study addressed anxiety but not depression in children and adolescents aged 8–18 years in Germany (42).

Three studies included a pre-pandemic comparison measure of anxiety (5, 36, 39), and found that symptoms increased at the first assessment after onset of the COVID-19 pandemic in spring/summer 2020 (5, 36), or remained stable early in the pandemic and increased later on, i.e., in June 2021 (39).

Trends found for children’s anxiety symptoms were largely similar to what was observed for depression. The majority of studies (*n* = 7 studies set in the Netherlands, Norway, Germany, Canada, and China) indicated an increasing trend during the pandemic (5, 39, 42, 47, 51) or found that levels of anxiety symptoms were higher than before the pandemic both early on and later in the pandemic, though they slightly decreased between early and late measurement points (36). Several studies also found that anxiety levels peaked during times of high infection rates/lockdowns (5, 36, 42, 51, 56).

Zuccolo et al. (55) measured mental health outcomes 14 times in a large sample of children (ages 5–17 years) in Brazil between June 2020 and June 2021. They found an increase of anxiety in July 2020, followed by a decrease from October 2020 to February 2021, which coincided with a reduction in social distancing requirements in Brazil in late 2020, followed by another decrease from April to May 2021. They reported no pre-pandemic data (55).

In agreement with what they found for depressive symptoms, two of the Chinese studies noted a decrease in anxiety symptoms (48, 49).

#### Other internalizing symptoms

Ten studies reported results for internalizing symptoms assessed with instruments not specifically targeting depression or anxiety, but using most commonly the SDQ.

Lehmann et al. (35) analyzed data from the large Norwegian COVID-19 Young study including participants aged 11–19 years. They noted a significant increase in internalizing problems between the lockdown in April/May 2020 and 9 months later in Dec 2020/Jan 2021 (35). In a smaller sample of US children with data from the same study period (T1: April/May 2020, T2: Nov 2020-Jan 2020), the investigators also found that internalizing problems (SDQ) increased significantly during the pandemic (52). Another small United States study following participants with a mean age of 14.1 years came to the same conclusion and additionally noted that adolescent mental health was closely linked to maternal mental health (50). Similarly, the German COPSY study found that internalizing symptoms (peer and emotional problems) steadily increased in children and adolescents during the pandemic’s high infection and lockdown phases (T1: May/Jun 2020 to T2: Dec 2020/Jan 2021, also compared to pre-pandemic) and plateaued at the last assessment (T3: Sep/Oct 2021), where restrictions had been lifted again (5). A small sample of Israeli mothers provided information about young children’s conduct and emotional problems at four time points during the pandemic, namely September 2020 (lockdown), October 2020 (post-lockdown) and in January 2021 (lockdown) and March 2021 (post-lockdown) (54). They similarly found that emotional problems in the 2–5-year-old children were the highest during the first lockdown period (T1) and significantly decreased in the post-lockdown periods. Contrary to these studies, the PROMEHS study on adolescents aged 11–16 years in Italy, Latvia, and Portugal found no significant changes in adolescents’ internalizing (and externalizing) symptoms between October 2020 and May 2021 (40), and a Japanese study in preschoolers and school-aged children also noted no significant change in SDQ scores over time (46).

The large Dutch NTR (Netherlands Twin Register) study assessed internalizing symptoms in 8–18-year-old before and at two time points during the pandemic (Apr-May 2020, strict lockdown; Nov-Dec 2020, partial lockdown) (36). They found significantly higher levels of internalizing symptoms during the COVID-19 pandemic than before, but more so during the strict lockdown, with a decrease during the following partial lockdown (36). The WHISTLER study (Wheezing Illnesses Study Leidsche Rijn), also conducted in the Netherlands, and followed a small sample of adolescents from March 2019 (T0) to February 2021 (T4) (43). They found increased internalizing symptoms only during the second full lockdown at T4 (43). The large Tyrolean COVID-19 Children’s Study examined the effects of the pandemic and factors influencing the mental health and quality of life of children aged 3–13 in North Tyrol (Austria) and South Tyrol (Italy) at four different time points [Mar 2020 (lockdown), Dec 2020, Jun 2021, and Dec 2021]. The study found that mental health outcomes, including internalizing problems and posttraumatic stress symptoms, gradually increased and were worse in December 2021 compared to during lockdown in March 2020 in all age groups (44). Lastly, the aforementioned study in Brazil examined emotional problems in a large sample of children and adolescents aged 5–17 years and found that the total emotional problems increased in July and September 2020, decreased from December 2020 to February 2021, and then increased again in May 2021, compared to June 2020 (55). Despite these fluctuations, the authors reported no sustained increase.

Studies on internalizing symptoms, be it depression, anxiety or a broader mental health construct, mostly conclude that symptom levels in children and adolescents increased or remained high during the pandemic not only compared to before, but also many months or even over a year after the onset of the pandemic, oftentimes in relation to periods with pandemic restrictions such as lockdowns or school closures.

### Mental health—externalizing symptoms

Externalizing disorders include Attention-Deficit/Hyperactivity Disorder (ADHD), Conduct Disorder (CD), Oppositional Defiant Disorder (ODD), and Antisocial Personality Disorder (ASPD). Eight studies assessed externalizing symptoms during the pandemic (5, 35, 38, 40, 44, 46, 50, 54), oftentimes considering maternal mental health as well. Gordon-Hacker et al. (54) report that children’s conduct problems were highest during the second lockdown (September 2020) in Israel and dropped in the post-lockdown periods. A similar trend was found by Cimino et al. (38), who examined mother–child dyads in Italy (children ages 5–6), with pre-pandemic data from Oct 2019 and two pandemic follow-ups in Mar 2020 (lockdown) and Oct 2021 (post-lockdown). They compared children of mothers with a high risk of psychopathology and those with a low risk. Interestingly, the authors found that in the no-risk group, symptoms of aggression in the children increased significantly between 2019 and 2020, but significantly decreased again by the assessment in 2021, when there was no more lockdown, reaching even lower levels than in 2019. In the high-risk group however, aggression decreased from 2019 to 2020 and again in 2021 (38). Another study assessed the relation between adolescent and maternal mental health early in the pandemic (April 2020) compared to 6 months later in a small sample of US adolescents. While studying adolescents’ mental health trajectories was not the main objective, the results indicated an increase in externalizing problems over time, which was strongly predicted by maternal mental health (50).

Martinsone et al. (40) describe a sample of adolescents from the PROMEHS study (Latvia, Italy, and Portugal), assessed in October 2020 and May 2021, and their caregivers. This study found no changes in externalizing difficulties scores between these time points, during a period characterized by strict COVID restrictions and high mortality rates (40). Another longitudinal study in Japanese parents of children aged 0–9 years also found no significant changes in externalizing symptoms throughout different stages of the pandemic between lockdown in March 2020 and February 2021, in the investigated group of children aged 4–9 years (46). While the Norwegian COVID-19 Young study found an overall increase in internalizing difficulties, there was no significant change in the level of externalizing symptoms, with conduct problems as well as hyperactivity remaining stable between April/May 2020 and December 2020/January 2021 (35).

The aforementioned COPSY study describes a significant increase in externalizing symptoms, specifically from pre-pandemic levels to May/June 2020 (first lockdown in Germany). The percentage of children with abnormal symptoms significantly increased from a pre-pandemic 13% to approximately 18% for conduct and 22% for hyperactivity problems during the first lockdown. These rates remained elevated throughout December 2020/January 2021 and September/October 2021 (5). Similar results have been reported by an Austrian group finding a significant pandemic-related increase in aggressive behavior according to longitudinal data collected from a large sample of parents (44).

Studies on externalizing symptoms paint a more heterogeneous picture than the results found for internalizing symptoms. While two studies found an increase in externalizing symptoms during the pandemic (44, 50), three studies found no change (35, 40, 46). Another study also noted no change during the pandemic, but had pre-pandemic data suggesting higher levels at all times during the pandemic (5). Lastly, two studies noted a decrease during the pandemic, after lockdown, but had pre-pandemic data suggesting that levels of externalizing symptoms had initially increased during lockdown (38, 54).

### Health-related quality of life

Three large (*n* > 1,000) studies (5, 34, 37) and one smaller study (41) investigated changes in HRQoL during the pandemic. All of these studies used data from 2020 to 2021, three of them included pre-pandemic data as well (5, 37, 41). The studies were set in Germany (*n* = 2), Switzerland, and Norway. They all used a version of the KIDSCREEN to assess HRQoL, and the current pandemic restriction measures such as lockdowns were considered in the interpretation of the results.

The German LIFE Child study investigated changes in KIDSCREEN scores in the domains of physical well-being, psychological well-being, and peers and social support in 9–16-year-old German children, covering a 10-month-follow-up period during the pandemic (41). Compared to before the pandemic, all domains decreased during the first lockdown, and physical well-being had further decreased by the second lockdown, while there was no change in the domains peers and social support (41). The German COPSY study found similar trends in a general population sample of children and adolescents (5). The percentage of participants with poor HRQoL increased significantly from 15% pre-pandemic to 40% in T1 (May/Jun 2020, end first lockdown 2020) and 48% in T2 (Dec 2020/Jan 2021, second lockdown 2020), and improved slightly to 35% in T3 (Sep/Oct 2021, loosened restrictions)—though this rate is still more than double the pre-pandemic percentage (5). Albrecht et al. (37) investigated overall HRQoL in a large sample of Swiss high school students at two times during the pandemic (during school closure in April 2020 and 12 months later, post-closure) and compared this data with a pre-pandemic control group. HRQoL was significantly better in the closure group and lower in the post-closure group compared to the control group.

The Norwegian COVID-19 Young study (34) investigated HRQoL during (Apr/May 2020) and 9 months after the national lockdown (Dec 2020/ Jan 2021) and, consistent with the findings of Poulain et al. (41), found a significant decline of physical and psychological well-being between these time points. Peer and social support, however, increased over time while the other domains of HRQoL (autonomy and parent relations; school environment) showed no change.

In summary, the three studies with pre-pandemic data observed a decrease in HRQoL that coincided with the first lockdown in Germany and Switzerland (5, 37, 41). The longitudinal evidence of the identified studies suggests that decreases in HRQoL of children and adolescents persist months, or even over a year, after the start of the pandemic and related lockdown measures, and persist further even when restrictions are no longer in place.

### Gender differences

Fifteen of the studies analyzed gender differences in mental health and/or HRQoL during the COVID-19 pandemic, with mixed results. Three studies found no gender differences in mental health outcomes (38, 48) and no differential change in symptoms between boys and girls (54). One study found no differential change in symptoms, but a higher mental health symptom load in girls (43). In another study, male gender predicted aggressive behavior, but there was no significant association of gender with internalizing symptoms and PTSD symptoms (44). Altogether, 10 studies indicated that girls had higher levels of internalizing symptoms (5, 40, 42, 43, 47, 49, 51, 55, 56) and/or a more pronounced increase in internalizing symptoms than boys (39, 47).

Regarding HRQoL, one study found that girls had lower initial HRQoL and a steeper decline in HRQoL over time than boys (34), and similarly, a second study found higher proportions of girls with low HRQoL, and high anxiety and depressive symptoms both before and during the pandemic (5).

## Discussion

This systematic review investigated the development of child and adolescent mental health and HRQoL throughout the COVID-19 pandemic based on published literature identified in a thorough systematic search. To our knowledge, this is the first systematic review to collect empirical evidence on COVID-19-related trajectories of mental health and quality of life in children and adolescents, focusing on population-based studies that cover at least 6 months of pandemic time and at least two pandemic assessment points. Building on recent evidence comparing pre-pandemic and pandemic data that has shown a decrease in young people’s mental health during the COVID-19 pandemic, such as the systematic review by Kauhanen et al. (22) or the scoping review by Wolf and Schmitz (26), this study addresses the question of how children’s and adolescents’ mental health and HRQoL has developed over the course of the pandemic, and especially beyond the first lockdowns.

In total, 24 prospective studies were included in the final review. Most of the studies investigated mental health outcomes, with a strong focus on internalizing symptoms such as anxiety and depression. Fewer studies (*n* = 4) investigated children’s HRQoL during the pandemic. Overall, the quality of evidence we found was predominantly moderate or low according to risk of bias assessment (Table 1) and notably, only few studies included large and representative samples (5, 36, 39, 56).

The core result of this synthesis is that, despite some heterogeneity in the results, most of the evidence suggests an increase in young people’s mental health problems and poor quality of life during the pandemic, also beyond the initial phase of lockdowns.

Since this comparison was not the focus of this review, not all included studies had pre-pandemic outcome data. However, the comparison with pre-pandemic data has been covered by previous reviews showing substantial evidence that mental health in young people has decreased compared to before the pandemic (15, 22). Results of a meta-analyses showed that pooled prevalence estimates of clinically-elevated depression and anxiety symptoms in children and adolescents during the first year of the pandemic were 25.2 and 20.5%, respectively, which implies that the respective prevalence has doubled compared to pre-pandemic estimates (4).

The results of this review indicate that the burden of mental health problems and decreased HRQoL has further increased, or at the very least remained elevated, throughout the pandemic years 2020 and 2021 in many countries. Fluctuations in symptom levels were often attributed to phases with strict restriction measures. In particular, the strength of the restriction measures varied greatly not only between the 16 countries examined in this study but also within each country, making a comparison difficult. Some studies, however, noted no changes or even noticed an improvement in mental health outcomes over time. Inconsistencies in findings among the reviewed studies may be due to the variability in study samples, such as different assessment times, contexts and country/region. These variations are connected to differences in infection rates, (strength of) health protection measures, and the duration and intensity of exposure to the pandemic at the time of assessment. The timing of assessments might be a significant factor when studying changes in symptoms throughout the pandemic.

Interestingly, the studies covering 12 or more months of the pandemic and using large, representative samples, such as the COPSY study (5), the Tyrolean COVID-19 Children’s Study (44), and the UEVO study (39), found that mental health symptoms and decreased HRQoL persisted or continued to increase, even when strict restrictions or lockdowns were no longer in place. The results of this review suggest that having experienced the COVID-19 crisis with all its implications for public and family life might have long-term effects on the mental health and well-being of the young population, and we might consequently face an accumulated need for youth mental health services and support after the pandemic. It is possible that the reason some studies did not observe a decrease in symptoms after lifting restrictions is that recovery takes time and may not be immediately noticeable in assessments. Even without restrictions, certain stressors like the unpredictability of the situation and fear of infection have likely continued to impact mental health.

The reviewed literature shows that externalizing symptoms and HRQoL were far less frequently studied in a longitudinal design than internalizing symptoms such as depression and anxiety. Externalizing symptoms also appear to be impacted by the pandemic to a lesser degree. There might be several hypothetical explanations for this finding. One important factor could be that a strong majority of the studies focused on older children and adolescents, where externalizing symptoms are less common than in children of preschool age or younger, while internalizing symptoms increase in adolescence (58). The results of the studies in this review might indicate that externalizing symptoms are not as strongly affected by social isolation, in fact, in some studies they even decreased, underpinning the fact that these symptoms are of a highly heterogeneous origin and also have a strong genetic component (59).

For the internalizing symptoms, the observed results, mainly indicating an increase during the pandemic and peaks during phases of high restrictions, appear plausible. Decreased peer contacts, school closures, fear of infection, and the disruption of family life are known exacerbators of anxiety and depression symptoms that have been previously discussed in the literature (18). In terms of risk factors and pathways to mental health and well-being, the included studies described a variety of environmental and also pandemic-related factors that also played a role in the level of mental health symptoms and HRQoL during the pandemic. Among these were peer and family conflict, parenting practices, previous psychiatric diagnosis, parental psychopathology, socioeconomic disadvantages, and reduced social contact. However, analyzing these risk factors in detail is beyond the scope of this review. Though, we did examine and summarize whether studies reported gender differences. We found that 12/15 studies examining gender reported poorer mental health and well-being and/or steeper declines during the pandemic for girls particularly for internalizing symptoms. This is consistent with the current state of evidence, which has demonstrated that girls tend to have a higher load of internalizing symptoms than boys (60) and that girls’ and women’s mental health and well-being appear to have been affected more by the pandemic (6). Concerning risk and protective factors (other than gender) for mental health problems during the COVID-19 pandemic, the recent scoping review by Wolf and Schmitz provides an excellent overview (26). Their synthesis suggests that low socioeconomic status, financial worries, material hardship, lack of space, negative home-schooling experience, poor physical health, and pre-existing neurodevelopmental disorders represent key risk factors for experiencing more pronounced negative mental health effects during the pandemic (26).

Even though the pandemic has evidently affected children’s and adolescents’ mental health and well-being, research shows there are several resilience factors in young people and families (26) that could be strengthened through interventions in the future, particularly targeting the most vulnerable groups of children. One example is the promotion of physical activity, which can mitigate the negative effects of the COVID-19 pandemic by improving young people’s moods (61, 62). A recent meta-analysis has also demonstrated that psychosocial interventions that enable personal interaction and include a physical activity component showed greater effectiveness in improving children’s and adolescents’ mental health outcomes (63). Future research should also focus on monitoring other health outcomes relevant to the young demographic, such as eating disorders. According to an analysis of administrative data from the largest German statutory health insurance, there has been a significant increase in hospital admissions for anorexia nervosa among children and adolescents during the pandemic, particularly among girls (64). This suggests that crises like the pandemic, which involve social isolation and school closures, can aggravate eating disorders in young people, possibly due to increased social media activity. Thus, even though many children and adolescents show resilience in times of crisis, this vulnerable group should not be forgotten.

### Strengths and limitations

A primary strength of this systematic review is that it encompassed 24 studies, collectively examining a substantial number of children and adolescents across 16 countries. These papers provided valuable longitudinal data regarding the development of various mental health issues and measures of HRQoL throughout the pandemic. Synthesizing these findings, this study is addressing an important research gap.

However, this review also has certain limitations. Due to the nature of the study question, the heterogeneous study designs, the different assessment points, and the diverse outcomes assessed by the reviewed studies, a quantitative synthesis using meta-analysis was deemed inappropriate. Instead, data was extracted, visualized, and narratively synthesized to a very detailed extent (see Table 1 and Figure 2). However, the presence and strength of the restriction measures between and within the included countries could not be addressed in detail due to the large number of countries and high variations. Furthermore, the inclusion criteria were restricted to peer-reviewed papers, potentially resulting in the omission of relevant information published at pre-print stage or grey literature like governmental reports or reports from insurance providers. We only included peer-reviewed publications to ensure the methodological quality of the studies. Despite the inclusion of 24 studies, many of them were small or based on non-representative data, thereby limiting the generalizability of the findings. Further, studies from African countries were missing, limiting our conclusions mainly to a European context, and there was a dearth of studies examining children below school age, which limited the interpretability of findings for this specific age group. As for the outcome measures, it can be positively noted that most studies used validated instruments. However, it needs to be emphasized that such instruments commonly used to screen for mental health symptoms such as anxiety or depression are not suited to (categorically) diagnose any mental disorders but rather to assess population-level trends in symptom load. Lastly, as with any systematic review, there is the risk of publication bias, which could for example lead to an overestimation of effects if non-results were systematically less frequently published.

### Conclusion

The results of this systematic review point toward a sustained increase in mental health problems, particularly internalizing symptoms such as anxiety and depression, and a reduced quality of life in children and adolescents during the first 2 years of the COVID-19 pandemic. The identified studies were heterogeneous regarding the studied populations and methods applied, and high-quality evidence from large, representative population samples was scarce.

From a public health point of view, these results point toward the importance of preventing mental health problems in children and adolescents. Promoting mental health and well-being, especially in times of crisis and especially in particularly vulnerable groups, is important in order to prevent symptoms of, for example, anxiety and depression from becoming manifest disorders that might persist into adulthood.

It is critical to continue monitoring children and adolescents to learn about their mental health and well-being after the pandemic, preferably on a broad, collaborative scale, in representative samples, and using validated instruments. This requires a systematic approach, such as national research networks, and, ideally, the use of the same instruments, which would facilitate a comparison between countries.

As the results demonstrate long-term consequences of the COVID-19 pandemic, the mental health and well-being of children and adolescents requires a stronger consideration in the future context of pandemic management, especially when considering the implementation of strict measures such as school closures and lockdowns.

## Author contributions

EO: Writing – original draft, Conceptualization, Data curation, Formal analysis, Investigation, Methodology, Visualization. LL: Conceptualization, Data curation, Formal analysis, Investigation, Methodology, Writing – review & editing. MG: Data curation, Formal analysis, Writing – review & editing. A-KN: Data curation, Formal analysis, Writing – review & editing. AK: Conceptualization, Project administration, Supervision, Writing – review & editing. ST: Methodology, Project administration, Writing – review & editing. MB: Methodology, Writing – review & editing. JD: Writing – review & editing. FR: Writing – review & editing. FW: Data curation, Formal analysis, Writing – review & editing. CJ-S: Data curation, Formal analysis, Writing – review & editing. VE: Data curation, Formal analysis, Writing – review & editing. MF: Data curation, Formal analysis, Writing – review & editing. EM: Formal analysis, Writing – review & editing. EB: Data curation, Writing – review & editing. SB: Investigation, Writing – review & editing. UR-S: Conceptualization, Funding acquisition, Investigation, Project administration, Resources, Supervision, Validation, Writing – review & editing.

## Footnotes

^1^ www.rayyan.ai
