# Supplementary material for: Mental health and quality of life in children and adolescents during the COVID-19 pandemic: a systematic review of longitudinal studies
Source: Front Public Health. 2024 Jan 8;11:1275917. doi: 10.3389/fpubh.2023.1275917 (PMC10800626; doi:10.3389/fpubh.2023.1275917)
Supplement: Supplementary file 1 [file Data_Sheet_1.DOCX]

**Supplemental Material**

**Appendix 1: Database search strategy**

| **Ovid MEDLINE(R**) and In-Process, In-Data-Review & Other Non-Indexed Citations and Daily <1946 to August 29, 2022> | | |
| --- | --- | --- |
| # |  | results |
| 1 | (Infant* or baby or babies or child* or toddler* or adolescent* or teen* or minors or youth).tw. | 2088621 |
| 2 | (mental or well*being or depress* or anxiety* or anxious or quality of life or QoL or HRQoL or mood or psych* or stress* or emotion* or behavior* or behaviour* or externali* or internali*).tw. | 3861412 |
| 3 | exp mental health/ or exp mental disorders/ | 1426015 |
| 4 | (covid* or coronavirus or corona virus or pandemic* or sars-cov-2).tw. | 303497 |
| 5 | exp COVID-19/ or exp Coronavirus/ or exp SARS-CoV-2/ | 198196 |
| 6 | (longitudinal or long*term or longer-term or prospective* or trajector* or cohort* or repeat* or panel* or survey* or ((measure* or assess* or collect*) adj4 points) or wave*).tw. | 3468929 |
| 7 | 1 and (2 or 3) and (4 or 5) and 6 | 2759 |
| 8 | limit 7 to yr="2020 -Current" | 2660 |

| **# Web of Science Search Strategy (v0.1)** |  |  |  |  |  |  |
| --- | --- | --- | --- | --- | --- | --- |
| # Database: Web of Science Core Collection |  |  |  |  |  |  |
| # Entitlements: |  |  |  |  |  |  |
| - WOS.SSCI: 1956 to 2022 |  |  |  |  |  |  |
| - WOS.AHCI: 1975 to 2022 |  |  |  |  |  |  |
| - WOS.CCR: 1985 to 2022 |  |  |  |  |  |  |
| - WOS.BHCI: 2009 to 2022 |  |  |  |  |  |  |
| - WOS.ISTP: 2009 to 2022 |  |  |  |  |  |  |
| - WOS.ESCI: 2005 to 2022 |  |  |  |  |  |  |
| - WOS.SCI: 1945 to 2022 |  |  |  |  |  |  |
| - WOS.BSCI: 2009 to 2022 |  |  |  |  |  |  |
| - WOS.ISSHP: 2009 to 2022 |  |  |  |  |  |  |
| - WOS.IC: 1993 to 2022 |  |  |  |  |  |  |
| # Searches: |  |  |  |  |  |  |
| 2: TS=((Infant$ or baby or babies or child* or toddler$ or adolescent$ or teen* or minors or youth) and (covid* or (corona NEAR/1 virus) or pandemic$ or sars-cov-2) and (mental or well$being or depress* or anxiet* or anxious or quality of life or QoL or HRQoL or mood or psych* or stress* or emotion* or behavior* or behaviour* or externali* or internali*) AND (longitudinal or long$term or longer-term or prospective* or trajector* or cohort* or repeat* or panel$ or survey* or ((measure* or assess* or collect*) NEAR/3 points) or wave*)) and 2020 or 2021 or 2022 or 2023 (Publication Years) | Editions: WOS.SCI,WOS.SSCI |  |  | Date run: Tue Aug 30 2022 13:20:57 GMT+0200 (Mitteleuropäische Sommerzeit) |  | Results: 3663 |

| **APA PsycInfo** <1806 to August Week 4 2022> | | |
| --- | --- | --- |
| # |  | results |
| 1 | (Infant* or baby or babies or child* or toddler* or adolescent* or teen* or minors or youth).tw. | 1025435 |
| 2 | (mental or well*being or depress* or anxiety* or anxious or quality of life or QoL or HRQoL or mood or psych* or stress* or emotion* or behavior* or behaviour* or externali* or internali*).tw. | 2759792 |
| 3 | exp mental disorders/ or exp mental health/ | 999823 |
| 4 | (covid* or coronavirus or corona virus or pandemic* or sars-cov-2).tw. | 24061 |
| 5 | exp coronavirus/ or exp covid-19/ | 14407 |
| 6 | (longitudinal or long*term or longer-term or prospective* or trajector* or cohort* or repeat* or panel* or survey* or ((measure* or assess* or collect*) adj4 points) or wave*).tw. | 765423 |
| 7 | 1 and (2 or 3) and (4 or 5) and 6 | 1100 |
| 8 | limit 7 to yr="2020 -Current" | 1063 |

| **PSYNDEXplus** Literature and Audiovisual Media <1977 to July 2022> | | |
| --- | --- | --- |
| # |  | results |
| 1 | (Infant* or baby or babies or child* or toddler* or adolescent* or teen* or minors or youth).mp. | 69850 |
| 2 | (mental or well*being or depress* or anxiety* or anxious or quality of life or QoL or HRQoL or mood or psych* or stress* or emotion* or behavior* or behaviour* or externali* or internali*).mp. | 277081 |
| 3 | exp mental disorders/ or exp mental health/ | 78509 |
| 4 | (covid* or coronavirus or corona virus or pandemic* or sars-cov-2).mp. | 1438 |
| 5 | exp covid-19/ or exp coronavirus/ | 700 |
| 6 | (longitudinal or long*term or longer-term or prospective* or trajector* or cohort* or repeat* or panel* or survey* or ((measure* or assess* or collect*) adj4 points) or wave*).mp. | 33979 |
| 7 | 1 and (2 or 3) and (4 or 5) and 6 | 68 |
| 8 | limit 7 to yr="2020 -Current" | 68 |

**Appendix 2: EPHPP QUALITY ASSESSMENT CHECKLIST****^[[1]](#footnote-1)^ adapted^[[2]](#footnote-2)^**

**A) SELECTION BIAS**

**A1 Are the individuals selected to participate in the study likely to be representative of the target population?**

Consider questions from the NICE guideline^[[3]](#footnote-3)^:

- Was the method of selection of participants from the eligible population well described?
- Were the inclusion or exclusion criteria explicit and appropriate?

Criteria: description of sampling method, description of sampling setting, (e.g., in-/exclusion criteria, recruitment sites and procedure), sample size

1. Very likely
2. Somewhat likely
3. Not likely
4. Can’t tell (selection not described)

**A2 What percentage of selected individuals agreed to participate?**

1. 80–100% agreement
2. 60–79% agreement
3. Less than 60% agreement
4. Not applicable*
5. Can’t tell**

*Not applicable: Consider whether it has been described in the paper, why it was not possible to report a response rate.

**Can’t tell: if the response rate was not mentioned AND it’s not possible to calculate the response rate based on reported statistics in the paper.

**RATE SECTION A**

**1 Strong**: The selected individuals are very likely to be representative of the target population (A1 is 1) and there is greater than 80% participation (A2 is 1) or the missing participation rate has been justified well (A2 is 4 with justification).

**2 Moderate**: The selected individuals are at least somewhat likely to be representative of the target population (A1 is 1 or 2) and there is 60–79% participation (A2 is 2). ‘Moderate’ may also be assigned if A1 is 1 or 2 and A2 is 4 (without justification).

**3 Weak**: The selected individuals are not likely to be representative of the target population (A1 is 3); or there is less than 60% participation (A2 is 3) or selection is not described (A1 is 4) and the level of participation is not described (A2 is 5).

**SELECTION BIAS**

**Low** The sample is very likely to be representative of the target population or a specific subgroup. The participation rate is either greater than 80% or well justified for not reporting (e.g., recruitment through flyers or posts on the internet).

**Moderate** The sample is at least somewhat likely to be representative of the target population or a specific subgroup. The participation rate is either between 60–79 % or the recruitment procedure shows indications of justification for not reporting.

**High** The sample is not likely to be representative of the target population or a specific subgroup. The participation rate is less than 60 %.

**B) STUDY DESIGN**

**B1 Indicate the type of longitudinal study design:**

1. Longitudinal study with multiple observations of (mainly) the same subjects, with or without topping up the sample
2. Repeated cross-sectional study where study participants are largely or entirely different on each sampling occasion
3. Can't tell

**B2 Were follow-up times / time points of data collection sufficiently described?**

Is it clear, when the respective data collections took place?

1. Yes
2. No
3. Can't tell

**RATE SECTION B**

**1 Strong**: Study follows the same participants over a well described period of time (B1 is 1 and B2 is 1)

**2 Moderate**: Study is longitudinal, but the follow-up time / time points aren’t sufficiently described (B1 is 1 and B2 is 2 or 3) or study is repeated cross-sectional with clearly described time points ((B1 is 2 and B2 is 1)

**3 Weak**: The study is repeated cross-sectional or unclear design with insufficiently described follow-up period (B1 is 2 or 3 and B2 is 2 or 3)

**E) DATA COLLECTION METHODS**

Only consider the measurement tools on the outcome level (i.e., Mental Health and QoL).

Measurement tools must be described as reliable and valid. If ‘face’ validity or ‘content’ validity has been demonstrated, this is acceptable.

**E1 Were the outcomes measured in a valid way?**^[[4]](#footnote-4)^

1. Yes
2. No
3. Can’t tell

**E2 Were the outcomes measured in a reliable way?**3

1. Yes
2. No
3. Can’t tell

**RATE SECTION E**

**1 Strong**: The data collection tools have been shown to be valid (E1 is 1); and the data collection tools have been shown to be reliable (E2 is 1).

**2 Moderate**: The data collection tools have been shown to be valid (E1 is 1); and the data collection tools have not been shown to be reliable (E2 is 2) or reliability is not described (E2 is 3).

**3 Weak**: The data collection tools have not been shown to be valid (E1 is 2) or validity is not described (E1 is 3) or both reliability and validity are not described (E1 is 3 and E2 is 3).

**DETECTION BIAS**

**Low** Index variable was assessed through a validated and reliable instrument.

**Moderate** Index variable was assessed through a validated instrument which has not been shown to be reliable or its reliability is not described.

**High** The instrument used to assess index variable has not been shown to be valid. Or both reliability and validity are not described.

**F) WITHDRAWALS AND DROP-OUTS**

**F1 Were withdrawals and drop-outs (follow-up rate, or missing data in repeated cross-sectional studies) reported in terms of numbers and/or reasons?**

1. Yes
2. No
3. Can’t tell

**F2 Indicate the percentage of participants completing the study (follow-up rate, or missing data in repeated cross-sectional studies). (If the percentage differs by groups, record the lowest).**

1. 80–100%
2. 60–79%
3. Less than 60%
4. Can’t tell

The percentage of participants completing study and the percentage of missing data could also be indicated as reported sample size in the result section.

The combination of F1 = 2 und F2 = 1 is mostly rated in studies that did not report drop-outs or missing values but indicated in results that data from the whole sample has been used. In this case, it also qualifies for a moderate total rating.

**RATE SECTION F**

**1 Strong**: when the follow-up rate is 80% or greater (F1 is 1 and F2 is 1) or missing data was less than 20%.

**2 Moderate**: when the follow-up rate is 60 – 79% (F2 is 2) or missing data was 20–40% or F1 = 2 and F2 = 1 (see comment above).

**3 Weak**: when a follow-up rate is less than 60% (F2 is 3) or if the withdrawals and drop-outs were not described (F1 is 2 or F2 is 4) or missing data was more than 40% or missing data were not described.

**ATTRITION BIAS**

**Low** The follow-up rate is high, or percentage of missing data was low.

**Moderate** The follow-up rate is moderate, or percentage of missing data was tolerable.

**High** The follow-up rate is low, or percentage of missing data was high.

**H) ANALYSES**

**H1 Are the statistical methods appropriate for the study design?**

This refers to the whole statistical analysis of the study (rating on the study level, i.e., not only statistics that has been extracted for the review).

Due to the complexity of statistical analyses and related statistical conditions, this section is only rated based on descriptions of the respective study and common statistical approaches in the relevant research area.

1. Yes
2. No
3. Can’t tell

**H2 Were strategies to deal with confounding factors stated? ^[[5]](#footnote-5)^**

(e.g., sociodemographic data, age, gender)

1. Yes
2. No
3. Can’t tell

**RATE SECTION H**

**1 Strong**: will be assigned when H1 is 1 and H2 is 1.

**2 Moderate**: will be assigned when H1 is 2 and H2 is 1 OR H1 is 2 and H2 is 2.

**3 Weak**: will be assigned when H1 is 3 and H2 is 2 or 3.

**GLOBAL RATING**

**1 STRONG** (no WEAK ratings in the sections)

**2 MODERATE** (one WEAK rating in the sections)

**3 WEAK** (two or more WEAK ratings in the sections)

**Appendix 3: PRISMA Checklist**

| **Section and Topic** | **Item #** | **Checklist item** | **Location where item is reported** |
| --- | --- | --- | --- |
| **TITLE** | | |  |
| Title | 1 | Identify the report as a systematic review. | Title |
| **ABSTRACT** | | |  |
| Abstract | 2 | See the PRISMA 2020 for Abstracts checklist. | Abstract |
| **INTRODUCTION** | | |  |
| Rationale | 3 | Describe the rationale for the review in the context of existing knowledge. | Introduction, 3^rd^ + 4^th^ paragraph |
| Objectives | 4 | Provide an explicit statement of the objective(s) or question(s) the review addresses. | Introduction, last paragraph |
| **METHODS** | | |  |
| Eligibility criteria | 5 | Specify the inclusion and exclusion criteria for the review and how studies were grouped for the syntheses. | Methods, sections: “Eligibility criteria” and “Data extraction process and synthesis method” |
| Information sources | 6 | Specify all databases, registers, websites, organisations, reference lists and other sources searched or consulted to identify studies. Specify the date when each source was last searched or consulted. | Methods, section “Data sources and search strategy” |
| Search strategy | 7 | Present the full search strategies for all databases, registers and websites, including any filters and limits used. | Methods, section “Data sources and search strategy + Appendix 1 |
| Selection process | 8 | Specify the methods used to decide whether a study met the inclusion criteria of the review, including how many reviewers screened each record and each report retrieved, whether they worked independently, and if applicable, details of automation tools used in the process. | Methods, section “Study selection” |
| Data collection process | 9 | Specify the methods used to collect data from reports, including how many reviewers collected data from each report, whether they worked independently, any processes for obtaining or confirming data from study investigators, and if applicable, details of automation tools used in the process. | Methods, section “Data extraction process and synthesis method” |
| Data items | 10a | List and define all outcomes for which data were sought. Specify whether all results that were compatible with each outcome domain in each study were sought (e.g. for all measures, time points, analyses), and if not, the methods used to decide which results to collect. | Methods, section “Data extraction process and synthesis method” + Table 1 |
|  | 10b | List and define all other variables for which data were sought (e.g. participant and intervention characteristics, funding sources). Describe any assumptions made about any missing or unclear information. | Methods, section “Data extraction process and synthesis method” + Table 1 |
| Study risk of bias assessment | 11 | Specify the methods used to assess risk of bias in the included studies, including details of the tool(s) used, how many reviewers assessed each study and whether they worked independently, and if applicable, details of automation tools used in the process. | Methods, section “Risk of bias assessment” + Appendix 2 |
| Effect measures | 12 | Specify for each outcome the effect measure(s) (e.g. risk ratio, mean difference) used in the synthesis or presentation of results. | Methods, section “Eligibility criteria”, 5^th^ paragraph |
| Synthesis methods | 13a | Describe the processes used to decide which studies were eligible for each synthesis (e.g. tabulating the study intervention characteristics and comparing against the planned groups for each synthesis (item #5)). | Methods, section “Data extraction process and synthesis method” |
|  | 13b | Describe any methods required to prepare the data for presentation or synthesis, such as handling of missing summary statistics, or data conversions. | Not relevant, because no meta-analysis was conducted |
|  | 13c | Describe any methods used to tabulate or visually display results of individual studies and syntheses. | Methods, section “Data extraction process and synthesis method” + Table 1 |
|  | 13d | Describe any methods used to synthesize results and provide a rationale for the choice(s). If meta-analysis was performed, describe the model(s), method(s) to identify the presence and extent of statistical heterogeneity, and software package(s) used. | Methods, section “Data extraction process and synthesis method” |
|  | 13e | Describe any methods used to explore possible causes of heterogeneity among study results (e.g. subgroup analysis, meta-regression). | not applicable |
|  | 13f | Describe any sensitivity analyses conducted to assess robustness of the synthesized results. | not applicable |
| Reporting bias assessment | 14 | Describe any methods used to assess risk of bias due to missing results in a synthesis (arising from reporting biases). | Methods, section “Risk of bias assessment” + Appendix 2 |
| Certainty assessment | 15 | Describe any methods used to assess certainty (or confidence) in the body of evidence for an outcome. | not applicable |
| **RESULTS** | | |  |
| Study selection | 16a | Describe the results of the search and selection process, from the number of records identified in the search to the number of studies included in the review, ideally using a flow diagram. | Results, 1^st^ paragraph + Figure 1 |
|  | 16b | Cite studies that might appear to meet the inclusion criteria, but which were excluded, and explain why they were excluded. | not applicable |
| Study characteristics | 17 | Cite each included study and present its characteristics. | Results, 2^th^ to 5^th^ paragraph |
| Risk of bias in studies | 18 | Present assessments of risk of bias for each included study. | Results, 6^th^ paragraph + Table 2 |
| Results of individual studies | 19 | For all outcomes, present, for each study: (a) summary statistics for each group (where appropriate) and (b) an effect estimate and its precision (e.g. confidence/credible interval), ideally using structured tables or plots. | Table 1 |
| Results of syntheses | 20a | For each synthesis, briefly summarise the characteristics and risk of bias among contributing studies. | 1^st^ paragraph in subsection “Depression”, “Anxiety”, “Other internalising symptoms”, “Externalizing symptoms” and “Health-related quality of life” |
|  | 20b | Present results of all statistical syntheses conducted. If meta-analysis was done, present for each the summary estimate and its precision (e.g. confidence/credible interval) and measures of statistical heterogeneity. If comparing groups, describe the direction of the effect. | not applicable |
|  | 20c | Present results of all investigations of possible causes of heterogeneity among study results. | not applicable |
|  | 20d | Present results of all sensitivity analyses conducted to assess the robustness of the synthesized results. | not applicable |
| Reporting biases | 21 | Present assessments of risk of bias due to missing results (arising from reporting biases) for each synthesis assessed. | not applicable; reporting bias was included as part of the risk of bias assessment |
| Certainty of evidence | 22 | Present assessments of certainty (or confidence) in the body of evidence for each outcome assessed. | not applicable |
| **DISCUSSION** | | |  |
| Discussion | 23a | Provide a general interpretation of the results in the context of other evidence. | Discussion, starting 5^th^ paragraph |
|  | 23b | Discuss any limitations of the evidence included in the review. | Discussion, section “Strength and limitations”, 2^nd^ paragraph |
|  | 23c | Discuss any limitations of the review processes used. | Discussion, section “Strength and limitations”, 2^nd^ paragraph |
|  | 23d | Discuss implications of the results for practice, policy, and future research. | Conclusion, 2^nd^ to last paragraph |
| **OTHER INFORMATION** | | |  |
| Registration and protocol | 24a | Provide registration information for the review, including register name and registration number, or state that the review was not registered. | Abstract + Methods, 1^st^ paragraph |
|  | 24b | Indicate where the review protocol can be accessed, or state that a protocol was not prepared. | Methods, 1^st^ paragraph (Link to protocol is provided) |
|  | 24c | Describe and explain any amendments to information provided at registration or in the protocol. | not applicable |
| Support | 25 | Describe sources of financial or non-financial support for the review, and the role of the funders or sponsors in the review. | Funding |
| Competing interests | 26 | Declare any competing interests of review authors. | Conflict of interest |
| Availability of data, code and other materials | 27 | Report which of the following are publicly available and where they can be found: template data collection forms; data extracted from included studies; data used for all analyses; analytic code; any other materials used in the review. | Data Availability Statement“ |

*From:*  Page MJ, McKenzie JE, Bossuyt PM, Boutron I, Hoffmann TC, Mulrow CD, et al. The PRISMA 2020 statement: an updated guideline for reporting systematic reviews. BMJ 2021;372:n71. doi: 10.1136/bmj.n71

For more information, visit: <http://www.prisma-statement.org/>

1. https://www.ephpp.ca/quality-assessment-tool-for-quantitative-studies/ [↑](#footnote-ref-1)
2. Stuhrmann LY, Göbel A, Bindt C and Mudra S (2022) *Parental Reflective Functioning and Its Association With Parenting Behaviors in Infancy and Early Childhood: A Systematic Review*. Front. Psychol. 13:765312. doi: 10.3389/fpsyg.2022.765312 [↑](#footnote-ref-2)
3. National Institute for Health and Care Excellence (2012). *Methods for the development of NICE public health guidance* [Online]. Available: https://www.nice.org.uk/process/pmg4 [↑](#footnote-ref-3)
4. Wording from JBI Critical Appraisal Checklist for Cohort Studies: https://jbi.global/critical-appraisal-tools [↑](#footnote-ref-4)
5. Wording from JBI Critical Appraisal Checklist for Cohort Studies: https://jbi.global/critical-appraisal-tools [↑](#footnote-ref-5)
